# Elevated circulating FABP4 concentration predicts cardiovascular death in a general population: a 12-year prospective study

**DOI:** 10.1038/s41598-021-83494-5

**Published:** 2021-02-17

**Authors:** Norie Saito, Masato Furuhashi, Masayuki Koyama, Yukimura Higashiura, Hiroshi Akasaka, Marenao Tanaka, Norihito Moniwa, Hirofumi Ohnishi, Shigeyuki Saitoh, Nobuyuki Ura, Kazuaki Shimamoto, Tetsuji Miura

**Affiliations:** 1 Department of Cardiovascular, Renal and Metabolic Medicine, Sapporo Medical University School of Medicine, S-1, W-16, Chuo-ku, Sapporo, 060-8543 Japan; 2 Department of Cardiology, Kushiro Kojinkai Memorial Hospital, Kushiro, Japan; 3 Department of Public Health, Sapporo Medical University School of Medicine, Sapporo, Japan; 4 Department of Geriatric and General Medicine, Osaka University, Suita, Japan; 5 Department of Nursing, Division of Medical and Behavioral Subjects, Sapporo Medical University School of Health Sciences, Sapporo, Japan; 6 Sapporo Nishimaruyama Hospital, Sapporo, Japan; 7 Japan Health Care College, Sapporo, Japan

**Keywords:** Cardiovascular diseases, Metabolic disorders

## Abstract

Fatty acid-binding protein 4 (FABP4) is secreted from adipose tissue and acts as an adipokine, and an elevated circulating FABP4 level is associated with metabolic disorders and atherosclerosis. However, little is known about the causal link between circulating FABP4 level and mortality in a general population. We investigated the relationship between FABP4 concentration and mortality including cardiovascular death during a 12-year period in subjects of the Tanno-Sobetsu Study, a population-based cohort (n = 721, male/female: 302/419). FABP4 concentration at baseline was significantly higher in female subjects than in male subjects. All-cause death occurred in 123 (male/female: 74/49) subjects, and 34 (male/female: 20/14) and 42 (male/female: 26/16) subjects died of cardiovascular events and cancer, respectively. When divided into 3 groups according to tertiles of FABP4 level at baseline by sex (T1–T3), Kaplan–Meier survival curves showed that there were significant differences in rates of all-cause death and cardiovascular death, but not cancer death, among the groups. Multivariable Cox proportional hazard model analysis with a restricted cubic spline showed that hazard ratio (HR) for cardiovascular death, but not that for all-cause death, significantly increased with a higher FABP4 level at baseline after adjustment of age and sex. The risk of cardiovascular death after adjustment of age, sex, body mass index and levels of brain natriuretic peptide and high-sensitivity C-reactive protein in the 3rd tertile (T3) group (HR: 4.96, 95% confidence interval: 1.20–22.3) was significantly higher than that in the 1st tertile (T1) group as the reference. In conclusion, elevated circulating FABP4 concentration predicts cardiovascular death in a general population.

## Introduction

Fatty acid-binding proteins (FABPs), about 14–15-kDa cytosolic proteins, can bind long-chain fatty acids^1^ and have been proposed to facilitate the transport of lipids to specific organelles in the cell^1^. Among FABPs, fatty acid-binding protein 4 (FABP4), also known as adipocyte P2 (aP2) or adipocyte FABP (A-FABP), is expressed in adipose tissue including adipocytes and macrophages^1,2^. Previous studies using animal models showed that FABP4 contributes to the development of metabolic disorders and cardiovascular disease in communication with metabolic and inflammatory pathways in adipocytes and macrophages^3–5^. We previously showed in experimental models that inhibition of FABP4 by a small molecule might be a novel therapeutic strategy against insulin resistance, type 2 diabetes mellitus and atherosclerosis^6^. In a human study, subjects with a genetic variation of the FABP4 locus (T-87C) were shown to have a decrease in FABP4 expression in adipose tissue and beneficial effects on cardiovascular and metabolic health^7^.

FABP4 has no typical secretory signal peptides in the sequence of FABP4^1,2^, but secretion of FABP4 from adipocytes via a non-classical pathway has recently been confirmed^8,9^. Circulating FABP4 has been reported to act as an adipokine, an adipocyte-derived bioactive molecule, for the development of insulin resistance^8^ and atherosclerosis^10^ in experimental models. Furthermore, it has been reported that neutralization of secreted FABP4 with an antibody to FABP4 can be a novel strategy for treatment of insulin resistance, type 2 diabetes mellitus, atherosclerosis and vascular injury^8,11,12^.

Cross-sectional studies have shown that an elevated circulating FABP4 level is associated with obesity, insulin resistance, type 2 diabetes mellitus, hypertension, dyslipidemia, dysregulation of purine metabolism, atherosclerosis and disturbance of the heart, liver and kidney^13–19^. Longitudinal studies have also shown that FABP4 concentration can be a predictor for the development of metabolic syndrome^20^, type 2 diabetes mellitus^21^ and atherosclerosis^22^. Furthermore, FABP4 level has been reported to be associated with long-term cardiovascular events in patients with coronary heart disease^23^, type 2 diabetes mellitus^24^ and hemodialysis^25^. However, little is known about the causal link between circulating FABP4 level and mortality including cardiovascular death and cancer death as hard endpoints in a general population. In the present study, we investigated the association of FABP4 level with mortality during a 12-year period in subjects of a general population.

## Results

### Characteristics of the study subjects

Characteristics of the 721 recruited subjects (male/female: 302/419) are shown in supplementary Table S1. Mean age, body mass index (BMI) and waist circumference of the recruited subjects were 64 ± 14 years, 24.0 ± 3.9 and 85.3 ± 10.2 cm, respectively. FABP4 concentration was significantly lower in male subjects (median [interquartile range]: 16.9 [12.5–22.9] ng/mL) than in female subjects (23.4 [16.7–30.4] ng/mL).

Basal characteristics of male and female subjects divided by subgroups according to tertiles of FABP4 level at baseline are shown in Tables 1 and 2, respectively. There were significant differences in parameters, including age, BMI, waist circumference, blood pressure, blood urea nitrogen, creatinine, estimated glomerular filtration rate (eGFR), uric acid, triglycerides, insulin, homeostasis model assessment of insulin resistance (HOMA-R), hemoglobin A1c, brain natriuretic peptide (BNP) and high-sensitivity C-reactive protein (hsCRP), in both male and female subjects. In male subjects, there were significant differences in the use of a calcium channel blocker and thiazolidinedione among the groups (supplementary Table S2). In female subjects, there were significant differences in the use of an angiotensin II receptor blocker, calcium channel blocker, β blocker, diuretic, statin and eicosapentaenoic acid among the groups (Supplementary Table S3).

**Table 1:** Basal characteristics of the male subjects with tertiles of FABP4 level at baseline (n = 302).

Tertiles of FABP4 level	T1	T2	T3	*P*
	(5.3–14.2 ng/mL)	(14.2–21.0 ng/mL)	(21.2–50.6 ng/mL)	
	n = 100	n = 101	n = 101	
Age (years)	61 ± 14	65 ± 13*	69 ± 13*	< 0.001
Body mass index	23.2 ± 2.9	24.4 ± 2.6	25.5 ± 5.3*	< 0.001
Waist circumference (cm)	80.9 ± 8.6	85.7 ± 7.0*	89.3 ± 10.0*	< 0.001
Systolic blood pressure (mmHg)	134 ± 23	143 ± 21*	142 ± 20*	0.006
Diastolic blood pressure (mmHg)	78 ± 11	82 ± 11*	79 ± 12	0.028
Pulse rate (beats/min)	68 ± 9	66 ± 9	66 ± 10	0.616
Smoking habit	33 (33.0)	25 (24.8)	31 (30.7)	0.416
Alcohol drinking habit	32 (32.0)	35 (34.7)	44 (43.6)	0.204
**Comorbidity**				
Hypertension	42 (42.0)	62 (61.4)*	64 (63.4)*	0.004
Diabetes mellitus	11 (11.0)	11 (10.9)	25 (24.8)*	0.008
Dyslipidemia	41 (41.0)	48 (47.5)	55 (54.5)	0.161
**Biochemical data**				
AST (IU/L)	24 (20–29)	26 (21–34)	25 (21–31)	0.128
ALT (IU/L)	21 (16–28)	25 (19–32)	22 (15–34)	0.139
γGTP (IU/L)	25 (19–42)	30 (19–46)	32 (22–55)	0.073
Blood urea nitrogen (mg/dL)	16 ± 4	17 ± 4	18 ± 6*	0.007
Creatinine (mg/dL)	0.7 ± 0.1	0.8 ± 0.1*	0.9 ± 0.3*	< 0.001
eGFR (ml/min/1.73 m^2^)	89.1 ± 15.4	79.6 ± 15.5*	74.0 ± 21.3*	< 0.001
Uric acid (mg/dL)	5.6 ± 1.1	5.9 ± 1.1	6.4 ± 1.3*	< 0.001
Total cholesterol (mg/dL)	197 ± 36	192 ± 34	201 ± 36	0.249
LDL cholesterol (mg/dL)	122 ± 32	117 ± 33	122 ± 34	0.462
HDL cholesterol (mg/dL)	58 ± 14	54 ± 12	54 ± 16	0.060
Triglycerides (mg/dL)	81 (66–108)	100 (75–143)	108 (80–146)*	0.014
Fasting glucose (mg/dL)	101 ± 25	103 ± 21	108 ± 25	0.092
Insulin (µU/ml)	3.8 (2.7–5.4)	4.8 (3.4–7.5)	6.1 (4.0–10.3)*	< 0.001
HOMA-R	0.94 (0.64–1.45)	1.22 (0.82–1.83)	1.52 (0.92–2.95)*	< 0.001
Hemoglobin A1c (%)	5.2 ± 0.5	5.2 ± 0.6	5.4 ± 0.7*	0.012
BNP (pg/mL)	15 (8–23)	16 (10–36)	20 (10–38)*	0.038
hsCRP (mg/dL)	0.04 (0.02–0.07)	0.06 (0.03–0.12)	0.07 (0.04–0.12)*	0.025

Variables are expressed as number (%), means ± SD or medians (interquartile ranges).

*AST* aspartate transaminase, *ALT* alanine transaminase, *BNP* brain natriuretic peptide, *eGFR* estimated glomerular filtration rate, *FABP4* fatty acid-binding protein 4, *γGTP* γ-glutamyl transpeptidase, *HDL* high-density lipoprotein, *HOMA-R* homeostasis model assessment of insulin resistance, *hsCRP* high-sensitivity C-reactive protein, *LDL* low-density lipoprotein.

*P < 0.05 versus T1.

**Table 2:** Basal characteristics of the female subjects with tertiles of FABP4 level at baseline (n = 419).

Tertiles of FABP4 level	T1	T2	T3	*P*
	(6.4–18.4 ng/mL)	(18.4–27.3 ng/mL)	(27.4–85.5 ng/mL)	
	n = 139	n = 140	n = 140	
Age (years)	56 ± 15	65 ± 12*	70 ± 10*	< 0.001
Body mass index	21.3 ± 3.1	23.8 ± 3.0*	25.9 ± 4.2*	< 0.001
Waist circumference (cm)	78.2 ± 9.3	85.7 ± 9.4*	92.0 ± 9.4*	< 0.001
Systolic blood pressure (mmHg)	126 ± 25	136 ± 21*	147 ± 27*	< 0.001
Diastolic blood pressure (mmHg)	74 ± 13	77 ± 12	80 ± 13*	0.001
Pulse rate (beats/min)	69 ± 12	72 ± 12	71 ± 9	0.321
Smoking habit	22 (15.8)	10 (7.1)*	9 (6.4)*	0.013
Alcohol drinking habit	85 (61.2)	104 (74.3)*	109 (77.9)*	0.005
**Comorbidity**				
Hypertension	50 (36.0)	79 (56.4)*	94 (67.1)*	< 0.001
Diabetes mellitus	5 (3.6)	11 (7.9)	10 (7.1)	0.288
Dyslipidemia	37 (26.6)	79 (56.4)*	76 (54.3)*	< 0.001
**Biochemical data**				
AST (IU/L)	21 (18–24)	23 (19–27)*	25 (21–30)*	< 0.001
ALT (IU/L)	15 (13–19)	19 (14–24)*	20 (15–26)*	< 0.001
γGTP (IU/L)	16 (13–22)	18 (14–27)	20 (14–28)	0.103
Blood urea nitrogen (mg/dL)	13 ± 4	15 ± 4*	16 ± 4*	< 0.001
Creatinine (mg/dL)	0.5 ± 0.1	0.6 ± 0.1	0.7 ± 0.6*	0.003
eGFR (ml/min/1.73 m^2^)	91.5 ± 19.5	83.6 ± 15.7*	74.1 ± 19.4*	< 0.001
Uric acid (mg/dL)	4.3 ± 0.9	4.7 ± 0.9*	5.1 ± 1.2*	< 0.001
Total cholesterol (mg/dL)	199 ± 31	212 ± 31*	212 ± 35*	0.001
LDL cholesterol (mg/dL)	116 ± 28	127 ± 32*	129 ± 32*	0.001
HDL cholesterol (mg/dL)	67 ± 15	63 ± 17*	61 ± 16*	0.002
Triglycerides (mg/dL)	71 (56–100)	95 (67–122)*	94 (74–136)*	< 0.001
Fasting glucose (mg/dL)	92 ± 11	98 ± 19*	100 ± 22*	0.001
Insulin (µU/ml)	4.1 (3.0–5.5)	4.9 (3.7–7.0)*	5.9 (4.1–8.7)*	< 0.001
HOMA-R	0.90 (0.67–1.22)	1.16 (0.80–1.78)*	1.37 (0.94–2.29)*	< 0.001
Hemoglobin A1c (%)	4.9 ± 0.4	5.2 ± 0.6*	5.3 ± 0.7*	< 0.001
BNP (pg/mL)	17 (11–23)	19 (11–37)*	23 (14–40)*	0.003
hsCRP (mg/dL)	0.02 (0.01–0.05)	0.04 (0.02–0.08)	0.05 (0.03–0.10)*	< 0.001

Variables are expressed as number (%), means ± SD or medians (interquartile ranges).

*AST* aspartate transaminase, *ALT* alanine transaminase, *BNP* brain natriuretic peptide, *eGFR* estimated glomerular filtration rate, *FABP4* fatty acid-binding protein 4, *γGTP* γ-glutamyl transpeptidase, *HDL* high-density lipoprotein, *HOMA-R* homeostasis model assessment of insulin resistance, *hsCRP* high-sensitivity C-reactive protein, *LDL* low-density lipoprotein.

*P < 0.05 versus T1.

### Mortality and FABP4 level at baseline

The mean follow-up period was 10.4 years (range 0.1–12.2 years), and follow-up summation was 7512 (male/female: 3045/4467) person-years. The number of censored subjects other than death was 88, and the follow-up rate was 87.8%. During a 12-year period, all-cause death occurred in 123 (male/female: 74/49) subjects, and 34 (male/female: 20/14) and 42 (male/female: 26/16) subjects died of cardiovascular events and cancer, respectively. Causes of cardiovascular death included acute myocardial infarction (n = 3), sudden cardiac death (n = 2), heart failure (n = 12), stroke (n = 5), cardiovascular hemorrhage (n = 3) and other cardiovascular causes (n = 9). Organs of cancer in cases of cancer death included the lung (n = 13), stomach (n = 5), colon (n = 4), pancreas (n = 4) and others (n = 16). Cumulative incidences of all-cause death, cardiovascular death and cancer death were 17.1% (male/female: 24.5%/11.7%), 4.7% (male/female: 6.6%/3.3%) and 5.8% (male/female: 8.6%/3.8%), respectively.

**Figure 1:**
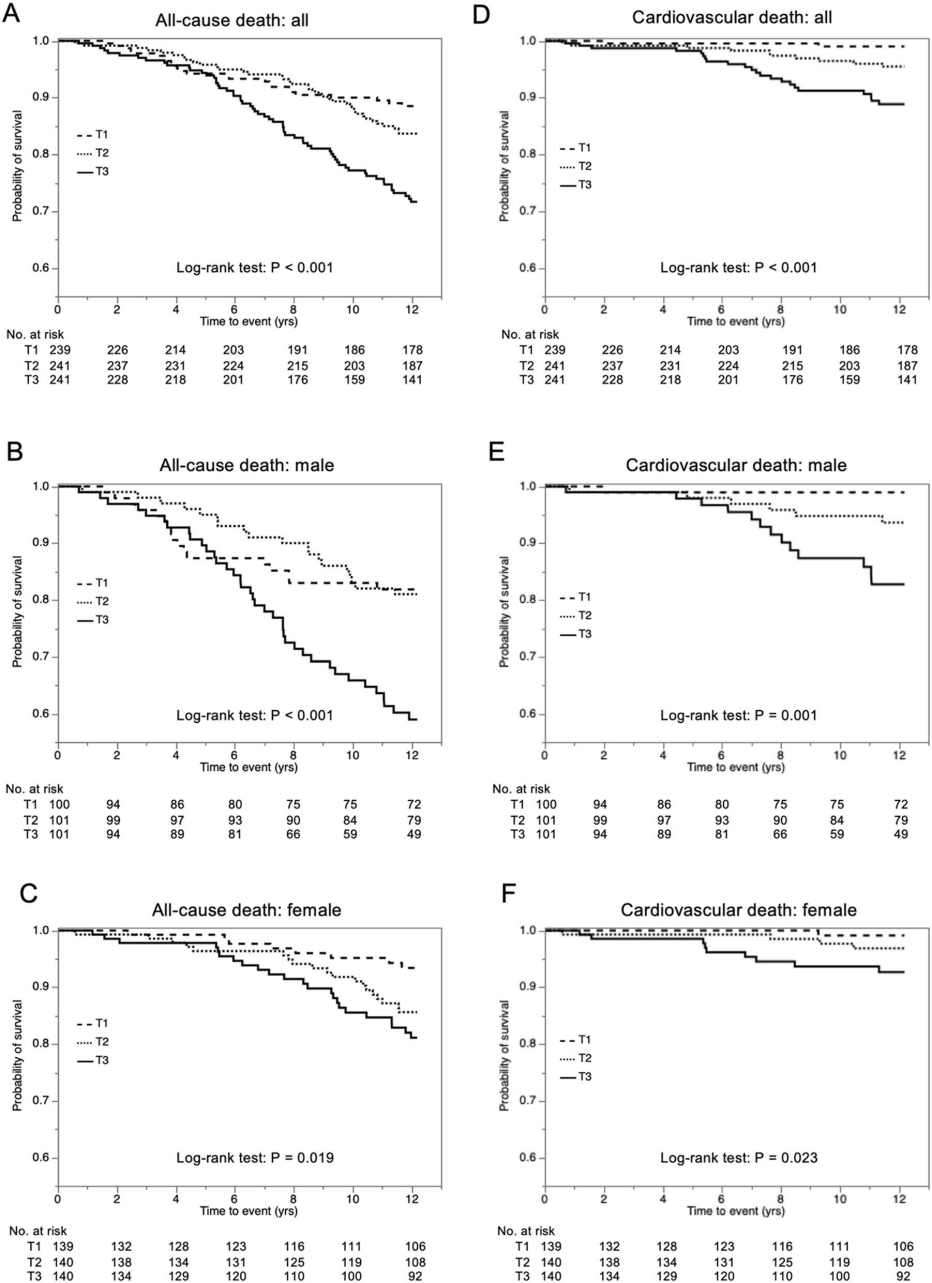
Survival curves of subgroups divided by tertiles of FABP4 level at baseline. (**A**–**C**) Kaplan–Meier survival curves for all-cause death in the three groups (T1–T3) according to tertiles of fatty acid-binding protein 4 (FABP4) concentration at baseline in all of the subjects (**A**) and in male (**B**) and female (**C**) subjects. (**D**–**F**) Kaplan–Meier survival curves for cardiovascular death in the three groups (T1–T3) according to tertiles of FABP4 concentration at baseline in all of the subjects (**D**) and in male (**E**) and female (**F**) subjects. Dashed line (T1), dotted line (T2) and solid line (T3). Survival rate was compared by the log-rank test.

When divided into 3 groups according to tertiles of FABP4 level at baseline (T1–T3), Kaplan–Meier survival curves showed that there were significant differences in rates of all-cause mortality among the groups in all of the subjects (log-rank test: *P* < 0.001) (Fig. 1A) as well as in male subjects (Fig. 1B) and female subjects (Fig. 1C) when divided by sex. Similarly, there were significant differences in rates of cardiovascular death (Fig. 1D–F), but not cancer death (supplementary Figure S1A–C), among the groups.

### Prediction of mortality by FABP4 level at baseline

Receiver operating characteristic (ROC) analyses for predicting all-cause death showed that the cut-off points of FABP4 concentration at baseline in male and female subjects were 18.6 ng/mL (sensitivity: 64.9%, specificity: 63.2%, area under the curve [AUC]: 0.63) and 25.3 ng/mL (sensitivity: 65.3%, specificity: 60.3%, AUC: 0.64), respectively (supplementary Figure S2A, B). ROC analyses for predicting cardiovascular death showed that the cut-off points of FABP4 concentration at baseline in male and female subjects were 19.1 ng/mL (sensitivity: 80.0%, specificity: 61.7%, AUC: 0.72) and 25.3 ng/mL (sensitivity: 85.7%, specificity: 58.8%, AUC: 0.73), respectively (supplementary Figure S2C, D).

### Impact of FABP4 level at baseline on mortality during the follow-up period

**Figure 2:**
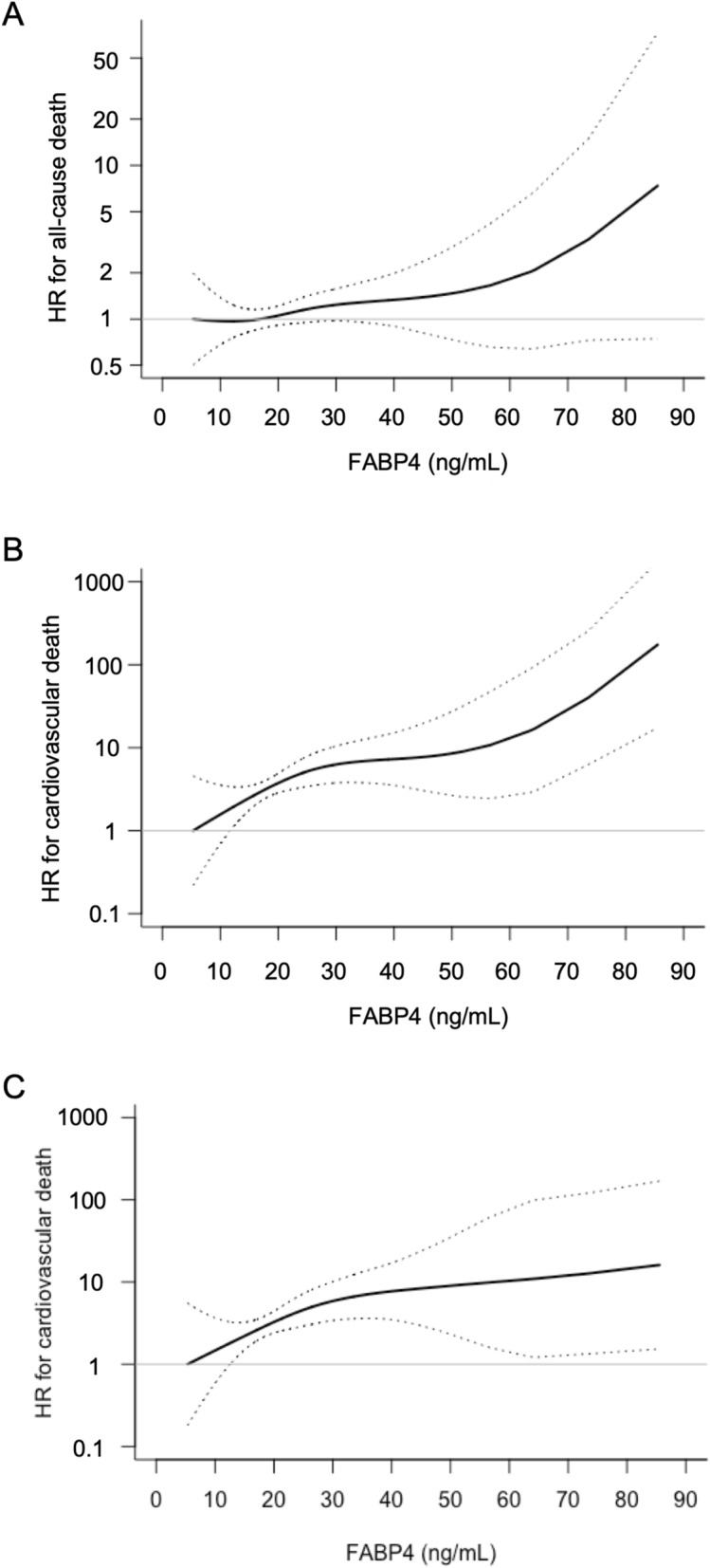
Hazard risk of FABP4 level for all-cause death and cardiovascular death. (**A**,**B**) Multivariable Cox proportional hazard analyses with a restricted cubic spline for all-cause death (**A**) and cardiovascular death (**B**) by fatty acid-binding protein 4 (FABP4) concentration at baseline after adjustment of age and sex during a 12-year follow-up period in all of the subjects. (**C**) Multivariable Cox proportional hazard analysis with a restricted cubic spline for cardiovascular death by FABP4 concentration at baseline after adjustment of age, sex, body mass index, brain natriuretic peptide and high-sensitivity C-reactive protein as the best-fit model during a 12-year follow-up period in all of the subjects. Solid line: hazard ratio (HR); dashed line: 95% confidence interval. The reference value of FABP4 was 5.3 ng/mL as a minimum value.

Multivariable Cox proportional hazard model analysis with a restricted cubic spline showed that hazard ratio (HR) for all-cause death after adjustment of age and sex tended to increase with a higher FABP4 level at baseline in all of the subjects (Fig. 2A), but the 95% confidence interval (CI) included the null value (HR = 1) calculated by a minimum value of FABP4 (5.3 ng/mL). The HR for cardiovascular death after adjustment of age and sex increased with a higher FABP4 level at baseline in all of the subjects (Fig. 2B), and the 95% CI did not include the null value (HR = 1) in most of the range of FABP4 levels.

Multivariable Cox proportional hazard model analysis after adjustment of age and sex showed that HR for cardiovascular death in the 3rd tertile (T3) group of FABP4 level was significantly higher than that in the 1st tertile (T1) group as the reference (Model 1, Akaike’s Information Criterion [AIC]: 384) (Table 3). There was no significant interaction between sex and FABP4 tertiles for cardiovascular death (*P* = 0.951). After adjustment of age, sex, BMI and BNP, the risk of cardiovascular death in the T3 group was significantly higher than that in the T1 group, and there was no significant interaction between sex and FABP4 tertiles (Model 2, AIC: 366). When hsCRP was additionally incorporated into Model 2, the risk of cardiovascular death in the T3 group (HR: 4.96, 95% CI 1.20–22.3) was significantly higher than that in the T1 group (Model 3, AIC: 323). When hsCRP, smoking habit and diagnosis of hypertension, diabetes mellitus and dyslipidemia at baseline were additionally incorporated into Model 2, the risk of cardiovascular death in the T3 group was significantly higher than that in the T1 group (Model 4, AIC: 329).

**Table 3:** Cox proportional hazard analyses for cardiovascular death in tertiles of FABP4 (n = 721).

	Model 1		Model 2		Model 3		Model 4	
	HR (95% CI)	*P*	HR (95% CI)	*P*	HR (95% CI)	*P*	HR (95% CI)	*P*
**FABP4**								
T1	Reference	–	Reference	–	Reference	–	Reference	–
T2	2.71 (0.59–12.5)	0.201	2.76 (0.59–12.9)	0.196	2.15 (0.44–10.5)	0.344	2.07 (0.42–10.2)	0.372
T3	4.98 (1.15–21.6)	0.032	5.38 (1.21–23.8)	0.028	4.96 (1.10–22.3)	0.037	4.83 (1.06–21.9)	0.041
Age (per 1 year)	1.08 (1.03–1.14)	< 0.001	1.09 (1.05–1.13)	< 0.001	1.09 (1.04–1.13)	< 0.001	1.08 (1.03–1.13)	0.001
Sex (male)	1.95 (0.68–5.62)	0.214	1.98 (0.69–5.71)	0.205	2.10 (0.71–6.27)	0.180	2.29 (0.76–6.89)	0.141
Interaction*		0.951		0.955		0.940		0.962
Body mass index	–	–	0.91 (0.86–0.97)	0.002	0.90 (0.85–0.97)	0.001	0.91 (0.86–0.98)	0.005
BNP (per 1 pg/mL)	–	–	1.01 (1.00–1.01)	< 0.001	1.01 (1.00–1.01)	< 0.001	1.01 (1.00–1.01)	< 0.001
hsCRP (per 1 mg/dL)	–	–	–	–	1.25 (0.01–49.4)	0.915	1.07 (0.01–41.7)	0.975
Smoking habit	–	–	–	–	–	–	0.85 (0.27–2.65)	0.778
Hypertension	–	–	–	–	–	–	1.89 (0.61–5.81)	0.267
Diabetes mellitus	–	–	–	–	–	–	0.44 (0.10–1.94)	0.281
Dyslipidemia	–	–	–	–	–	–	0.90 (0.42–1.96)	0.800
	(AIC = 384)		(AIC = 366)		(AIC = 323)		(AIC = 329)	

*Interaction: Sex—FABP4 tertiles.

*AIC* Akaike's information criterion, *BNP* brain natriuretic peptide, *CI* confidence interval, *FABP4* fatty acid-binding protein 4, *HR* hazard ratio, *hsCRP* high-sensitivity C-reactive protein.

The HR for cardiovascular death after adjustment of age, sex, BMI, BNP and hsCRP as the best-fit model using AIC increased with a higher FABP4 level at baseline in all of the subjects (Fig. 2C), and the 95% CI did not include the null value (HR = 1) in most of the range of FABP4 levels.

## Discussion

The present study showed the impact of elevated circulating FABP4 concentration on the incidence of cardiovascular mortality in a general population. FABP4 has been shown to play a significant role in the development of insulin resistance, type 2 diabetes mellitus and atherosclerosis through its action at the interface of metabolic and inflammatory pathways in adipocytes and macrophages^3–5^. Furthermore, previous studies using in vitro and in vivo experiments showed that secreted FABP4 acts as an adipokine and directly affects various types of cells including hepatocytes, macrophages, cardiomyocytes, vascular endothelial cells and vascular smooth muscle cells^8,10,12,26,27^, though potential receptors for FABP4 have still not been identified^2^. Therefore, FABP4 may contribute to classical risk factors including insulin resistance and/or metabolic syndrome and might be a kind of “master regulatory factor” of metabolic risk factors. Moreover, FABP4 per se may directly contribute to atherosclerosis and/or cardiovascular damage, independent of classical risk factors. The combination of these possibilities such as a key master regulatory factor and an independently direct action of FABP4 may be related to cardiovascular death.

It has been reported that FABP4 concentration can predict not only the development of metabolic syndrome^20^, type 2 diabetes mellitus^21^ and atherosclerosis^22^ but also long-term cardiovascular events in patients with coronary heart disease^23^, type 2 diabetes mellitus^24^ and hemodialysis^25^. Obesity is a risk factor of several metabolic and cardiovascular diseases^28^, and FABP4 concentration has been reported to be strongly associated with BMI^13,14^. It has also been reported that FABP4 level reflects myocardial lipid storage as a putative effector of cardiac damage^29^. Therefore, BMI as a marker of obesity and BNP as an indicator of cardiac damage were incorporated as confounders in Cox proportional hazard model analyses in the present study (Table 3). A community-based cohort study showed that FABP4 concentration was associated with cardiovascular events including cardiovascular death during a 12-year follow-up period, though the predictive value was not significant when hsCRP was added in the adjustment for traditional risk factors^30^. In the present study, we demonstrated that elevated circulating FABP4 concentration predicts cardiovascular death, but not all-cause death or cancer death, as a hard endpoint after adjustment of confounding factors including hsCRP in a general population (Table 3, Model 3 and Model 4). These observations support the notion that measurement of FABP4 concentration is useful for risk stratification and for guiding treatment in observational or interventional studies.

Secretion of FABP4 from adipocytes via a non-classical pathway is associated with lipolysis^8,9^. Secretion of FABP4 from macrophages as well as adipocytes has also been confirmed^10^, but the main source of FABP4 in blood circulation is adipocytes^5,8^. Lipolysis is more active in visceral fat than in subcutaneous fat^31^, and visceral obesity has been reported to promote oxidative stress^32^. FABP4 normally prefers to bind essential polyunsaturated fatty acids, linoleic acid and α-linolenic acid, but the affinity of FABP4 would be changed to prefer binding a saturated fatty acid, palmitic acid, due to conformational structure change of FABP4 in a condition of visceral obesity-induced oxidative stress^2,10^. It has been reported that palmitic acid-bound, but not fatty acid-unbound or linoleic acid-bound, FABP4 can be a deteriorating adipokine and induce inflammatory responses in several target cells, including macrophages, smooth muscle cells, endothelial cells, adipocytes and other cells^2,10^. However, potential receptors of FABP4 have not been identified yet, and there have been some reports about internalization of FABP4 in the cell and interaction of cytokeratin 1 with FABP4 on the endothelial cell membrane^2,12,33,34^. Increased visceral obesity and consumption of palmitic acid-rich foods may lead to an increase in palmitic acid-bound FABP4, resulting in the development of cardiovascular events.

FABP4-deficient mice have a strong compensatory increase of FABP5 in adipose tissue^3^. Mice with combined deficiency of FABP4 and FABP5 exhibited protection against type 2 diabetes, fatty liver disease and atherosclerosis more than did FABP4- or FABP5-deficient mice^35–37^. There have been no reports about longevity in FABP4-deficient or FABP5-deficient mice, but it has recently been shown that the lifespan of FABP4/5 double knockout mice was not longer than that of wild-type mice despite the advantage of metabolic health in FABP4/5 double knockout mice^38^. It has been reported that FABP4/5 double knockout mice had reduced cardiac contraction due to disturbed utilization of fatty acids in the heart in a model of pressure overload-induced cardiac hypertrophy and failure by transverse aortic constriction^39^. Ageing-induced damage in several tissues with a high demand for fatty acid metabolism may overcome the advantage of metabolic health in FABP4/5 double knockout mice, suggesting that deficiency of either FABP4 or FABP5 is better than a combined deficiency of FABP4 and FABP5 in adipose tissue in terms of longevity.

Several drugs, including angiotensin II receptor blockers^40,41^, a statin^42^, omega-3 fatty acid ethyl esters^43^, and dipeptidyl peptidase-4 (DPP-4) inhibitors^44,45^, have been reported to decrease circulating FABP4 level. Randomized controlled trials using these drugs except DPP-4 inhibitors showed improvement of cardiovascular outcomes^46–48^, and DPP-4 inhibitors have been reported to reduce intima media thickness, a marker of atherosclerosis^49,50^. Reduction of FABP4 level by drugs would be a novel therapeutic strategy for prevention and reduction of cardiovascular mortality. Recent studies using experimental models demonstrated the possibility of a new strategy for treating metabolic diseases including diabetes mellitus, atherosclerosis and vascular injury by targeting serum FABP4 with a monoclonal antibody to FABP4^8,11,12^. It is clearly necessary to prospectively evaluate whether a change in the FABP4 value by direct inhibition, neutralization and/or blockade of unidentified receptors indeed reflects conditions of metabolic syndrome and atherosclerosis and predicts long-term cardiovascular outcomes in the future.

The present study has several limitations. First, since the recruited subjects were only Japanese people, it is unclear whether the present findings can be generalized to other ethnicities. Second, recent studies have demonstrated modulation of FABP4 concentration by therapeutic drugs for hypertension, dyslipidemia and diabetes mellitus^40–45,51,52^, which might have affected cardiovascular events, though diagnosis of hypertension, dyslipidemia and diabetes mellitus was adjusted in Cox proportional hazard model analysis (Table 3, Model 4). Finally, FABP4 level was not investigated during the follow-up period. Investigation of the relationship between cardiovascular mortality and change in FABP4 level during a natural course and/or interventional studies would be needed in the future.

## Conclusion

Elevated circulating FABP4 concentration predicts cardiovascular death in a general population. FABP4 concentration may be not only a marker of metabolic disorders but also a predictor of cardiovascular mortality in a general population as well as in patients with metabolic and cardiovascular diseases. A further understanding of the mechanism underlying the link between circulating FABP4 and cardiovascular events may enable the development of new therapeutic strategies for metabolic and cardiovascular diseases such as inhibition of FABP4, neutralization of FABP4 and blockade of the possible FABP4 receptor.

## Methods

### Study subjects

In the Tanno-Sobetsu Study, a study with a population-based cohort, a total of 721 Japanese subjects (male/female: 302/419, mean age: 64 ± 14 years) were recruited from residents of Sobetsu Town who received annual health examination in 2007. This study was performed with the approval of the Ethical Committee of Sapporo Medical University and conformed to the principles outlined in the Declaration of Helsinki. Written informed consent was received from all of the study subjects.

### Follow-up and clinical outcome

All participants were followed up from 2007 to 2019, and their vital status, emigration status and cause of death were annually ascertained using residence registry data, death certificates, medical records in hospitals and/or questionnaires. Out-migrate individuals were defined as censored cases at the time of move-out day. The clinical endpoint was all-cause death including cardiovascular death and cancer death. Cardiovascular death included death resulting from acute myocardial infarction, sudden cardiac death and death due to heart failure, stroke, cardiovascular procedures, cardiovascular hemorrhage and other cardiovascular causes, as defined by the American Heart Association^53^.

### Measurements

Medical check-ups were performed early in the morning after an overnight fast as previously described^14^. Blood pressure was measured using an automated sphygmomanometer (HEM-907, Omron Co., Kyoto, Japan). BMI was calculated as body weight (kg) divided by the square of body height (meter). Peripheral venous blood samples were analyzed immediately or stored at − 80 °C until biochemical analyses.

Measurements of biochemical parameters were performed as previously reported^14^. The concentration of FABP4 was measured using a commercially available enzyme-linked immunosorbent assay kit for FABP4 (Biovendor R&D, Modrice, Czech Republic). The accuracy, precision and reproducibility of the kit have been described previously^13^. BNP was measured using an assay kit (Shionogi & Co., Osaka, Japan). hsCRP was measured by a nephelometry method.

Low-density lipoprotein (LDL) cholesterol level was calculated by the Friedewald equation^54^. Hemoglobin A1c (HbA1c) was expressed in National Glycohemoglobin Standardization Program (NGSP) scale. HOMA-R as an index of insulin resistance was calculated by the following^55^: HOMA-R = insulin (μU/mL) × glucose (mg/dL)/405. eGFR was calculated by an equation for Japanese^56^: eGFR (ml/min/1.73 m^2^) = 194 × creatinine^(−1.094)^ × age^(−0.287)^ × 0.739 (if female).

A self-administered questionnaire survey was performed to obtain information on smoking habit, alcohol drinking habit, and use of drugs for diabetes mellitus, hypertension and dyslipidemia. Hypertension was diagnosed as systolic blood pressure ≥ 140 mmHg, diastolic blood pressure ≥ 90 mmHg or self-reported use of anti-hypertensive drugs. Diabetes mellitus was diagnosed in accordance with the guidelines of the American Diabetes Association^57^: fasting plasma glucose ≥ 126 mg/dL, HbA1c ≥ 6.5% or self-reported use of anti-diabetic drugs. Dyslipidemia was diagnosed as LDL cholesterol ≥ 140 mg/dL, high-density lipoprotein (HDL) cholesterol < 40 mg/dL, triglycerides ≥ 150 mg/dL or self-reported use of anti-dyslipidemic drugs.

### Statistical analysis

Numeric variables are expressed as means ± standard deviation (SD) for normal distributions or medians (interquartile ranges) for skewed variables. The distribution of each parameter was tested for its normality using the Shapiro–Wilk W test. Intergroup differences in percentages of demographic parameters were examined by the chi-square test. Comparison between two groups was done with the Mann–Whitney's U test. One-way analysis of variance and Dunnett’s post hoc test were used for detecting significant differences in data between data in multiple groups. Survival rate was analyzed by the log-rank test of Kaplan–Meier curves. ROC analysis was performed to determine the inflection point at which the FABP4 level provided the most sensitive prediction of all-cause death and cardiovascular death in both male and female subjects, and the AUC of was calculated. The relationship between FABP4 concentration and hazard ratio (HR) for all-cause death, cardiovascular death or cancer death was analyzed by a multivariable Cox proportional hazard model with a restricted cubic spline after adjustment of age, sex and other confounders. In addition, the HRs and 95% CIs for cardiovascular death in three subgroups according to tertiles of FABP4 level at baseline (T1–T3) were calculated by adjustment of confounders including age, sex, BMI, smoking habit, levels of BNP and hsCRP, and diagnosis of hypertension, diabetes mellitus and dyslipidemia at baseline. Interaction of sex and tertiles of FABP4 level was also investigated. Among the candidate models, the best-fit model using AIC for each dependent variable was selected. A p value of < 0.05 was considered statistically significant. All data were analyzed by using JMP 14.3.0 for Macintosh (SAS Institute, Cary, NC) and R3.6.2.

## Supplementary Information

Supplementary Information

## Data Availability

The datasets analyzed during the current study are available from the corresponding author on reasonable request.
