# Supplementary material for: Elevated circulating FABP4 concentration predicts cardiovascular death in a general population: a 12-year prospective study
Source: Sci Rep. 2021 Feb 17;11:4008. doi: 10.1038/s41598-021-83494-5 (PMC7889640; doi:10.1038/s41598-021-83494-5)
Supplement: Supplementary file 1 — Supplementary Information [file 41598_2021_83494_MOESM1_ESM.pdf]

Supplementary materials

Elevated circulating FABP4 concentration predicts cardiovascular death in a general population: A 12-year prospective study.

Norie Saito, Masato Furuhashi, Masayuki Koyama, Yukimura Higashiura, Hiroshi Akasaka, Marenao Tanaka, Norihito Moniwa, Hirofumi Ohnishi, Shigeyuki Saitoh, Nobuyuki Ura, Kazuaki Shimamoto, Tetsuji Miura.

Figure S1

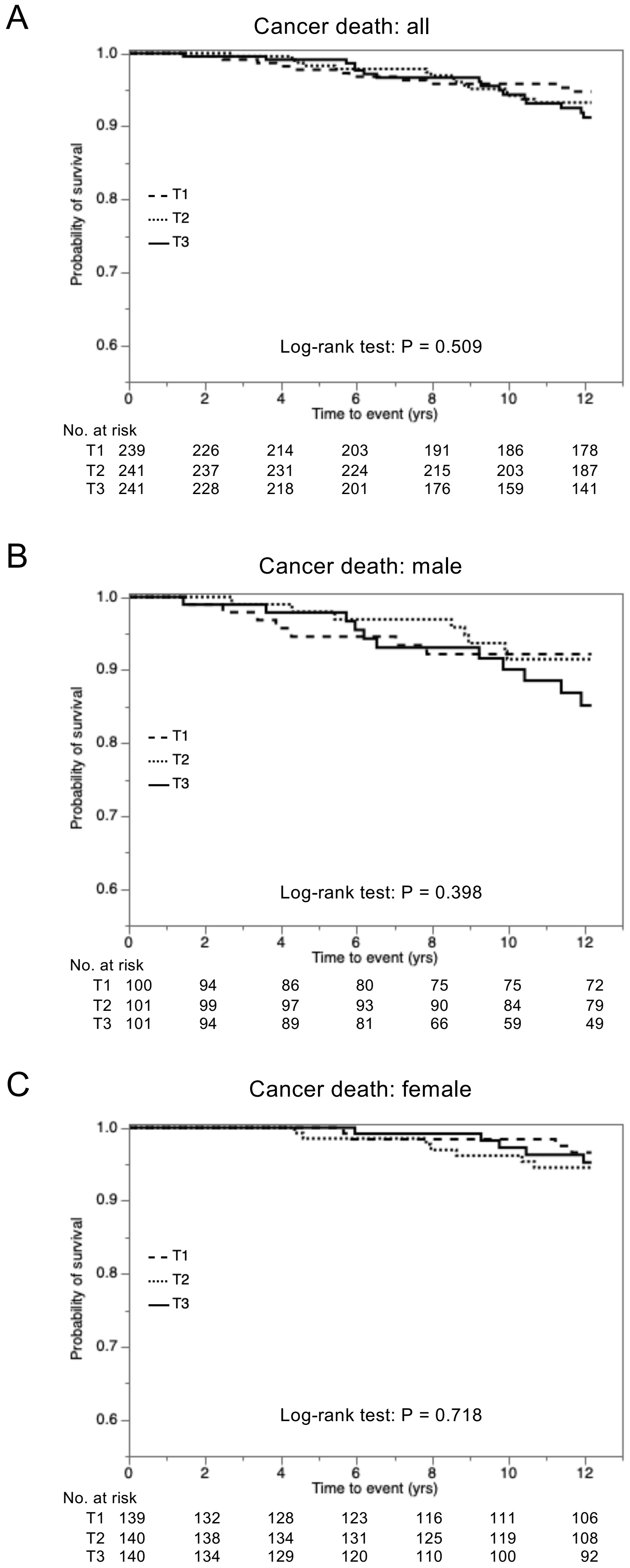

Figure S1. Survival curves of subgroups divided by tertiles of FABP4 level at baseline. A-C. Kaplan-Meier survival curves for cancer death in the three groups (T1~T3) according to tertiles of fatty acid-binding protein 4 (FABP4) concentration at baseline in all of the subjects (A) and in male (B) and female (C) subjects. Dashed line (T1), dotted line (T2) and solid line (T3). Survival rate was compared by log-rank test.

Figure S2

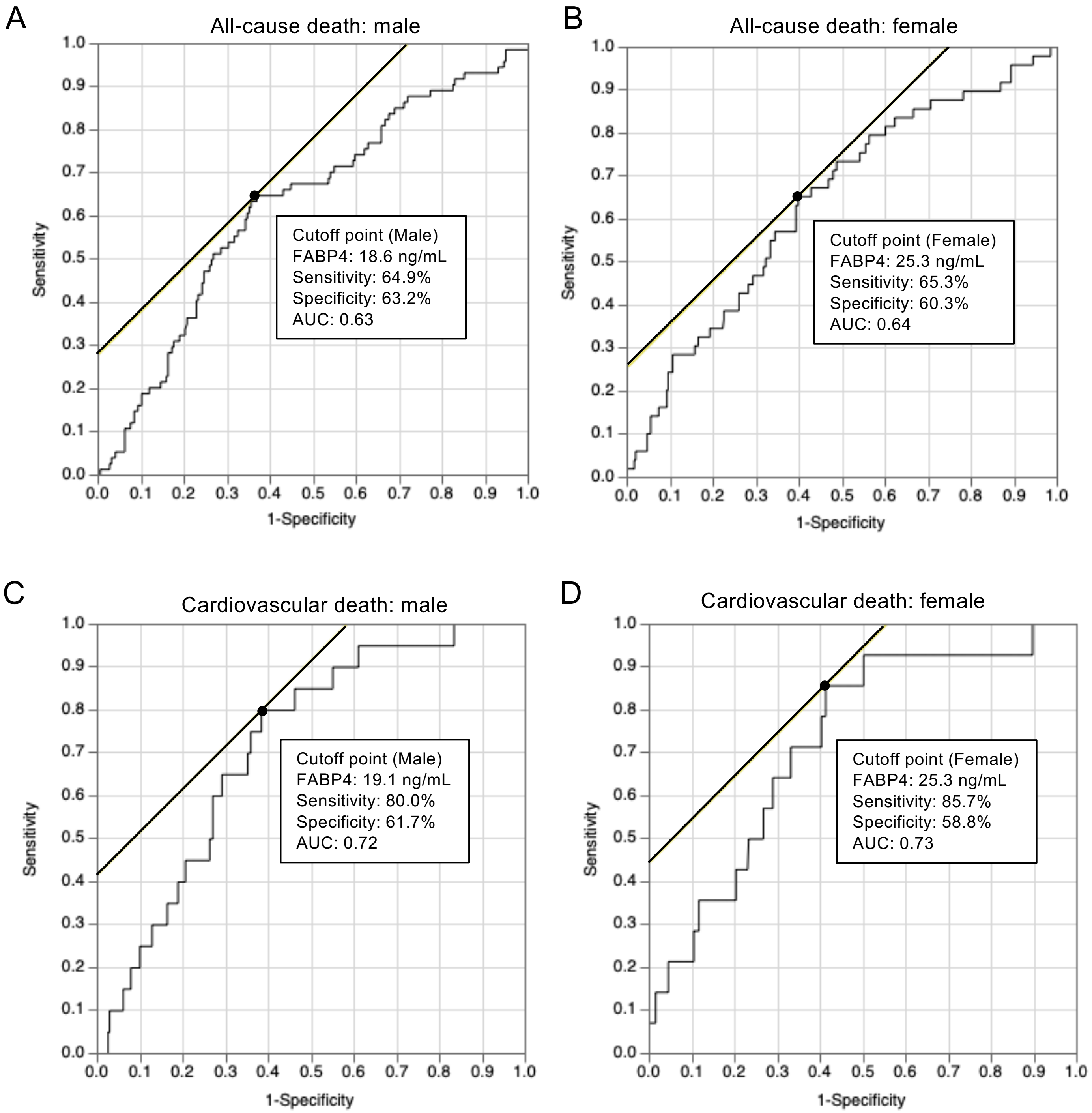

Figure S2. Prediction of mortality by FABP4 level at baseline.
A, B. Receiver operating characteristic (ROC) curves of fatty acid-binding protein 4 (FABP4) concentration at baseline to predict all-cause death in male (A) and female (B) subjects. **C, D.** ROC curves of FABP4 concentration at baseline to predict cardiovascular death in male (C) and female (D) subjects. For a given FABP4 level, the ordinate value shows the percentage of patients with that FABP4 level who died (true-positive rate or sensitivity), and the abscissa value shows the percentage of patients with that FABP4 level who did not die (false-positive rate or 1-specificty). AUC, area under curve.

Table S1

Table S1. Basal characteristics of the subjects (n = 721)

	Total<br>n = 721	Male<br>n = 302	Female<br>n = 419	P
Age (years)	64 ± 14	65 ± 14	64 ± 14	0.324
Body mass index	24.0 ± 3.9	24.4 ± 3.9	23.7 ± 3.9	0.021
Waist circumference (cm)	85.3 ± 10.2	85.3 ± 9.3	85.3 ± 10.9	0.999
Systolic blood pressure (mmHg)	138 ± 24	140 ± 22	137 ± 26	0.090
Diastolic blood pressure (mmHg)	78 ± 12	80 ± 11	77 ± 13	0.002
Pulse rate (beats/min)	69 ± 11	66 ± 9	71 ± 11	0.001
Smoking habit	130 (18.0)	89 (29.5)	41 (9.8)	< 0.001
Alcohol drinking habit	312 (43.3)	191 (63.2)	121 (28.9)	< 0.001
Comorbidity				
Hypertension	391 (54.2)	168 (55.6)	223 (53.2)	0.552
Diabetes mellitus	73 (10.1)	47 (15.6)	26 (6.2)	< 0.001
Dyslipidemia	336 (46.6)	144 (47.7)	192 (45.8)	0.622
Biochemical data				
AST (IU/L)	23 (20-29)	25 (20-30)	22 (19-27)	< 0.001
ALT (IU/L)	20 (15-27)	23 (17-31)	18 (14-24)	< 0.001
γGTP (IU/L)	21 (15-34)	30 (20-45)	18 (14-26)	< 0.001
Blood urea nitrogen (mg/dL)	16 ± 5	17 ± 5	15 ± 4	< 0.001
Creatinine (mg/dL)	0.7 ± 0.3	0.8 ± 0.2	0.6 ± 0.4	< 0.001
eGFR (ml/min/1.73m^2)	82.2 ± 19.2	80.9 ± 18.6	83.1 ± 19.6	0.129
Uric acid (mg/dL)	5.2 ± 1.3	6.0 ± 1.2	4.7 ± 1.1	< 0.001
Total cholesterol (mg/dL)	203 ± 34	197 ± 35	208 ± 33	< 0.001
LDL cholesterol (mg/dL)	122 ± 32	120 ± 33	124 ± 31	0.137
HDL cholesterol (mg/dL)	60 ± 16	55 ± 14	64 ± 16	< 0.001
Triglycerides (mg/dL)	91 (67-123)	98 (72-134)	86 (64-113)	< 0.001
Fasting glucose (mg/dL)	100 ± 21	104 ± 24	97 ± 19	< 0.001
Insulin (μU/ml)	4.7 (3.4-7.2)	4.7 (3.3-7.8)	4.8 (3.4-6.8)	0.760
HOMA-R	1.11 (0.79-1.83)	1.14 (0.78-1.94)	1.10 (0.79-1.76)	0.390
Hemoglobin A1c (%)	5.2 ± 0.6	5.3 ± 0.6	5.1 ± 0.6	0.001
BNP (pg/mL)	18 (11-33)	16 (9-32)	19 (12-34)	0.020
hsCRP (mg/dL)	0.04 (0.02-0.09)	0.05 (0.03-0.10)	0.04 (0.02-0.08)	< 0.001
FABP4 (ng/mL)	20.8 (14.5-27.6)	16.9 (12.5-22.9)	23.3 (16.7-30.4)	< 0.001

Variables are expressed as number (%), means ± SD or medians (interquartile ranges).
AST, Aspartate transaminase; ALT, Alanine transaminase; BNP, brain natriuretic peptide; eGFR, estimated glomerular filtration rate; FABP4, fatty acid-binding protein 4; γGTP, γ-glutamyl transpeptidase; HDL, high-density lipoprotein; HOMA-R, homeostasis model assessment of insulin resistance; hsCRP, high-sensitivity C-reactive protein; LDL, low-density lipoprotein.

Table S2

Table S2. Basal characteristics of the male subjects with tertiles of FABP4 level at baseline (n = 302)

Tertiles of FABP4 level	T1	T2	T3	P
n	100	101	101	
FABP4 level (ng/mL), range	5.3-14.2	14.2-21.0	21.2-50.6	
Medication				
Angiotensin II receptor blocker	7 (7.0)	5 (5.0)	10 (19.9)	3.965
Angiotensin-converting enzyme inhibitor	2 (2.0)	1 (1.0)	1 (1.0)	0.770
Calcium channel blocker	5 (5.0)	12 (11.9)	19 (18.8)*	0.010
β blocker	3 (3.0)	1 (1.0)	4 (4.0)	0.407
α blocker	1 (1.0)	0 (0.0)	3 (3.0)	0.171
Diuretic	1 (1.0)	0 (0.0)	3 (3.0)	0.171
Sulfonylurea	4 (4.0)	3 (3.0)	9 (8.9)	0.132
Biguanide	3 (3.0)	1 (1.0)	3 (3.0)	0.554
Thiazolidinedione	0 (0.0)	0 (0.0)	3 (3.0)*	0.049
α glucosidase inhibitor	4 (4.0)	1 (1.0)	5 (5.0)	0.260
Insulin	0 (0.0)	2 (2.0)	0 (0.0)	0.135
Statin	3 (3.0)	5 (5.0)	9 (8.9)	0.179
Fibrate	1 (1.0)	3 (3.0)	1 (1.0)	0.447
Eicosapentaenoic acid	2 (2.0)	1 (1.0)	4 (4.0)	0.362
Anti-platelet drugs	8 (8.0)	5 (5.0)	13 (12.9)	0.129
Warfarin	2 (2.0)	4 (4.0)	3 (3.0)	0.716

Variables are expressed as number (%), means ± SD or medians (interquartile ranges). *P < 0.05 vs. T1.
FABP4, fatty acid-binding protein 4.

Table S3

Table S3. Basal characteristics of the female subjects with tertiles of FABP4 level at baseline (n = 419)

Tertiles of FABP4 level	T1	T2	T3	P
n	139	140	140	
FABP4 level (ng/mL), range	6.4-18.4	18.4-27.3	27.4-85.5	
Medication				
Angiotensin II receptor blocker	4 (2.9)	13 (9.3)*	22 (15.7)*	0.001
Angiotensin-converting enzyme inhibitor	2 (1.4)	2 (1.4)	3 (2.1)	0.867
Calcium channel blocker	13 (9.4)	30 (21.4)*	31 (22.1)*	0.007
β blocker	0 (0.0)	3 (2.1)	6 (4.3)*	0.048
α blocker	0 (0.0)	1 (0.7)	0 (0.0)	0.368
Diuretic	2 (1.4)	1 (0.7)	8 (5.7)*	0.018
Sulfonylurea	1 (0.7)	5 (3.6)	5 (3.6)	0.228
Biguanide	0 (0.0)	3 (2.1)	3 (2.1)	0.221
Thiazolidinedione	0 (0.0)	0 (0.0)	0 (0.0)	-
α glucosidase inhibitor	2 (1.4)	2 (1.4)	3 (2.1)	0.867
Insulin	0 (0.0)	0 (0.0)	2 (1.4)	0.135
Statin	8 (5.8)	11 (7.9)	22 (15.7)*	0.013
Fibrate	1 (0.7)	2 (1.4)	2 (1.4)	0.820
Eicosapentaenoic acid	1 (0.7)	9 (6.4)*	5 (3.6)	0.037
Anti-platelet drugs	2 (1.4)	11 (7.9)	9 (6.4)	0.042
Warfarin	0 (0.0)	0 (0.0)	0 (0.0)	-

Variables are expressed as number (%), means ± SD or medians (interquartile ranges). *P < 0.05 vs. T1.
FABP4, fatty acid-binding protein 4.
